# A TNF Receptor 2 Selective Agonist Rescues Human Neurons from Oxidative Stress-Induced Cell Death

**DOI:** 10.1371/journal.pone.0027621

**Published:** 2011-11-14

**Authors:** Roman Fischer, Olaf Maier, Martin Siegemund, Harald Wajant, Peter Scheurich, Klaus Pfizenmaier

**Affiliations:** 1 Institute of Cell Biology and Immunology, University Stuttgart, Stuttgart, Germany; 2 Division of Molecular Internal Medicine, Department of Internal Medicine II, University Hospital Würzburg, Würzburg, Germany; Emory University, United States of America

## Abstract

Tumor necrosis factor (TNF) plays a dual role in neurodegenerative diseases. Whereas TNF receptor (TNFR) 1 is predominantly associated with neurodegeneration, TNFR2 is involved in tissue regeneration and neuroprotection. Accordingly, the availability of TNFR2-selective agonists could allow the development of new therapeutic treatments of neurodegenerative diseases. We constructed a soluble, human TNFR2 agonist (TNC-scTNF_R2_) by genetic fusion of the trimerization domain of tenascin C to a TNFR2-selective single-chain TNF molecule, which is comprised of three TNF domains connected by short peptide linkers. TNC-scTNF_R2_ specifically activated TNFR2 and possessed membrane-TNF mimetic activity, resulting in TNFR2 signaling complex formation and activation of downstream signaling pathways. Protection from neurodegeneration was assessed using the human dopaminergic neuronal cell line LUHMES. First we show that TNC-scTNF_R2_ interfered with cell death pathways subsequent to H_2_O_2_ exposure. Protection from cell death was dependent on TNFR2 activation of the PI3K-PKB/Akt pathway, evident from restoration of H_2_O_2_ sensitivity in the presence of PI3K inhibitor LY294002. Second, in an *in vitro* model of Parkinson disease, TNC-scTNF_R2_ rescues neurons after induction of cell death by 6-OHDA. Since TNFR2 is not only promoting anti-apoptotic responses but also plays an important role in tissue regeneration, activation of TNFR2 signaling by TNC-scTNF_R2_ appears a promising strategy to ameliorate neurodegenerative processes.

## Introduction

Tumor necrosis factor (TNF), plays a dominant role in the initiation and perpetuation of chronic inflammation [1], a condition that can lead to a variety of diseases. Blocking of TNF signaling has been evaluated in various inflammatory diseases and is successfully used for treatment of autoimmune diseases such as rheumatoid arthritis, Crohn's disease and psoriasis [2], [3]. Chronic inflammation is a common feature of neurodegenerative diseases of the central nervous system, such as Alzheimer's disease, Parkinson's disease (PD) and multiple sclerosis (MS), too [4], and TNF signaling has been implicated as an important factor for the onset of demyelinating diseases. However, despite promising results in mouse models of MS, clinical trials with TNF neutralizing reagents in MS patients failed to ameliorate the disease and in some cases even led to disease exacerbation [5].

Since then, the dual role of TNF in the CNS has been investigated in various mouse models, revealing significant beneficial effects predominantly mediated by TNF receptor (TNFR) 2, whereas TNFR1, directly and indirectly promotes neurotoxicity [2], [6]. In particular, it has been demonstrated that TNFR2 can protect neurons against excitotoxic insults *in vitro* [7], [8] and promotes neuronal survival as well as oligodendrocyte regeneration after ischemic and neurotoxic insults, respectively [9], [10]. In contrast, TNFR1 exacerbated axonal and neuronal damage through its potent pro-inflammatory effects, which became particularly obvious under chronic inflammatory situations [11]. Therefore, TNF remains a primary therapeutic target for the treatment of neuroimmune diseases, but therapeutic interference should be strictly receptor selective.

Using genetic engineering we have designed soluble human TNFR2-selective agonists. TNFR2 selectivity has been ensured by introducing known TNFR discriminating mutations in the TNF molecule (D143N/A145R; [12]). We used the TNFR2-selective mutant in the single-chain TNF format (scTNF_R2_), consisting of three TNF monomers connected by short peptide linkers [13], [14]. Since TNFR2 is only fully activated by membrane-bound TNF (memTNF) but not by soluble TNF trimers [15], the trimerization domain of tenascin C (TNC) was fused to the N-terminus of the TNFR2-selective scTNF. This molecule (TNC-scTNF_R2_) exists in a trimeric assembly of the single stranded fusion protein thus resembling a nonameric TNF molecule, which by its increased avidity mimics memTNF activity. In pilot studies with a prototype of TNC-scTNF_R2_ it has been verified that this molecule format allows activation of human TNFR2 without the requirement of secondary cross-linking of TNFR2 [16].

In the current work, we describe a fully human TNC-scTNF_R2_ in which peptide linker sequences were reduced and overall nucleic acid sequence was optimized for improved expression and function. After verifying selective human TNFR2 activation we evaluated the neuroprotective effect of TNC-scTNF_R2_ on LUHMES cells, an established neuronal precursor cell line that has retained the potential of neuronal differentiation into a dopaminergic phenotype [17], [18], [19], [20].

## Results

### Expression and characterization of human TNC-scTNF_R2_

**Figure 1:**
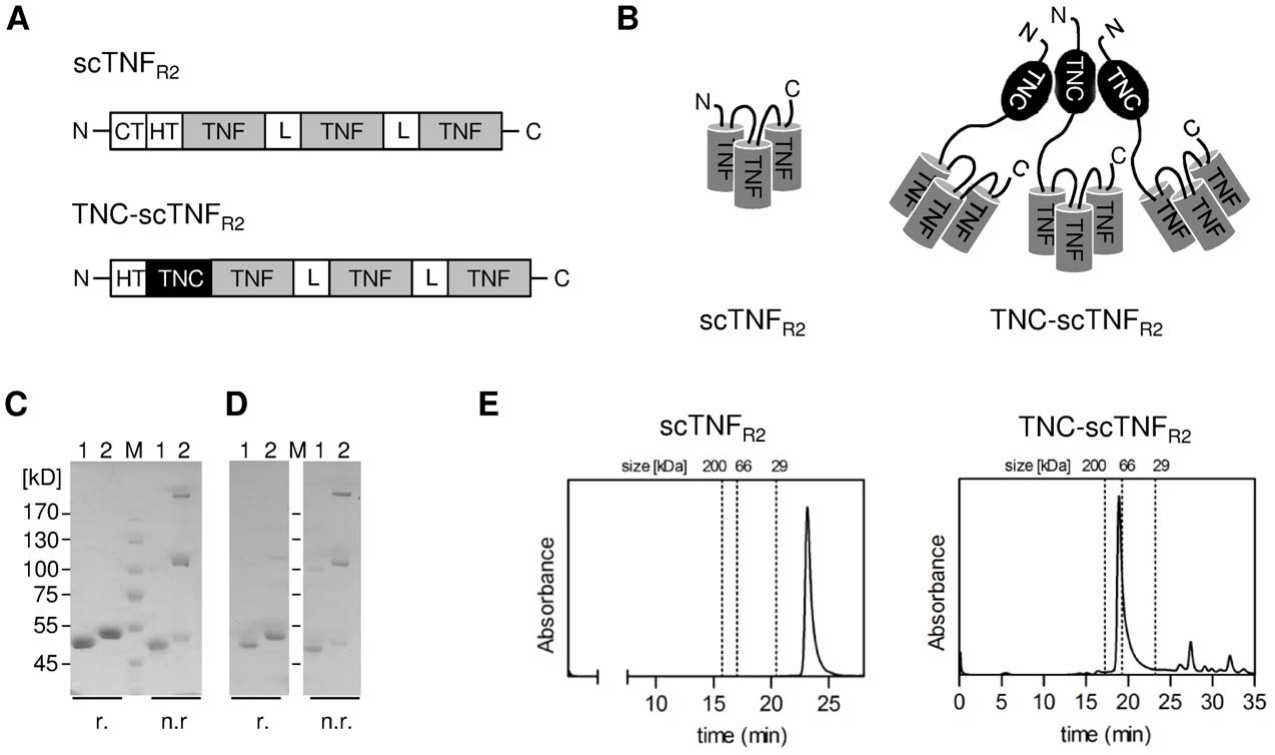
Genetic engineering of the TNFR2-selective TNF muteins. (**A,B**) Schematic representation of the TNF variants used in this study. CT: cys-tag, HT: his-tag; TNF: huTNFR2-specific (D143N/A145R) TNF module (aa 80–233); L: GGGGS-linker; TNC: trimerization domain of human tenascin C (aa 110–139). (**C**) Coomassie staining and (**D**) immunoblot analysis of scTNF_R2_ (1) and TNC-scTNF_R2_ (2). Purified TNF variants were analyzed by 8% SDS-PAGE under reducing (r.) or non-reducing (n.r.) conditions and either stained with Coomassie or immunoblotted with anti-his-tag antibodies. (**E**) TNF muteins were analyzed by HPLC size exclusion chromatography using a BioSep-Sec-2000 column. Peak positions of relevant standard proteins are indicated (200 kDa; 67 kDa and 29 kDa).

A prototype of a TNFR2-selective highly bioactive TNF variant has been recently reported [16], comprised of the chicken TNC trimerization domain and a TNFR2-selective TNF mutant (D143N/A145R; [12]) in the single chain TNF format [13]. To obtain a fully human TNF reagent we here fused the trimerization domain of human TNC (AA 110–139) to the N-terminus of the TNFR2-selective scTNF variant (TNC-scTNF_R2_), which was further modified for improved production. Thus, peptide linkers connecting the three TNF monomers were reduced to GGGGS and overall codon usage was adapted for expression in mammalian cell systems. Bioactivity of TNC-scTNF_R2_ was compared to the monomeric TNFR2-selective scTNF_R2_ (schematic overview in Fig. 1a and b).

Both scTNF_R2_ and TNC-scTNF_R2_ were expressed in HEK293T cells and isolated by immobilized metal ion chromatography (IMAC) in a single step using a N-terminal his-tag present in the molecule. Purity was confirmed by SDS-PAGE and Coomassie staining (Fig. 1C). Under reducing conditions the TNF variants exhibited an apparent molecular mass of approximately 51 kDa and 54 kDa, matching the calculated molecular mass of 53.5 kDa and 56.8 kDa for scTNF_R2_ and TNC-scTNF_R2_, respectively. Under non-reducing conditions additional bands of 110 kDa (dimer) and above 170 kDa (trimer) were observed for TNC-scTNF_R2_ (Fig. 1C and D). The oligomerization state of TNC-scTNF_R2_ was further characterized by size exclusion chromatography (SEC; Fig. 1E). Both scTNF_R2_ and TNC-scTNF_R2_ eluted as a single major peak and TNC-scTNF_R2_ with a higher molecular mass than scTNF_R2_, indicating that the TNC domain, as expected, causes stable oligomerization of scTNF_R2_ molecules. The fact that TNC-scTNF_R2_ elutes in a single peak while migrating in non-reducing SDS-PAGE as dimers and trimers is in good accordance with the finding that the TNC trimerization domain assembles into stable trimers without stabilization of disulfide bridges.

### Receptor selectivity and bioactivity of TNC-scTNF_R2_

**Figure 2:**
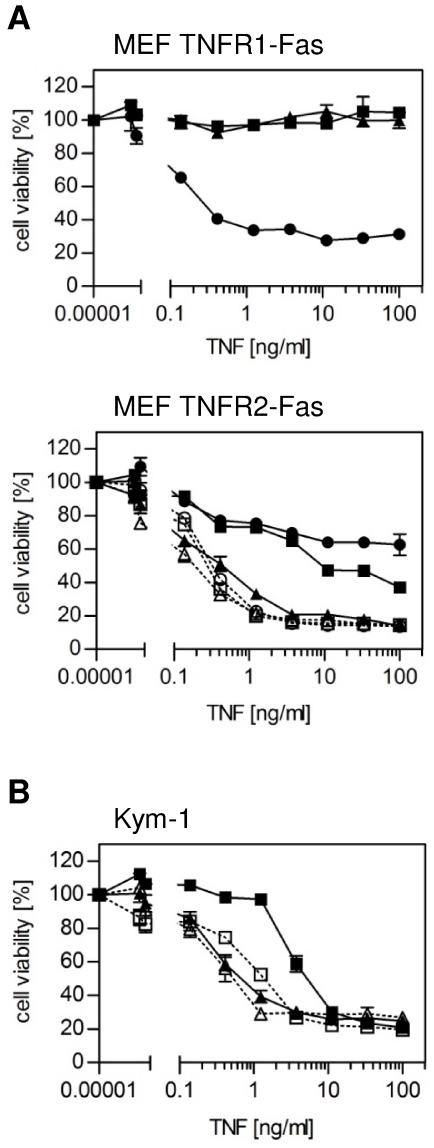
Bioactivity and receptor selectivity of the TNF muteins. Mouse embryonic fibroblasts (MEF) from TNFR1^−/−^/TNFR2^−/−^ mice stably transfected with the chimeric receptors TNFR1-Fas or TNFR2-Fas (**A**) or Kym-1 cells (**B**) were stimulated with wildtype human TNF (huTNF; •), scTNF_R2_ (▪) or TNC-scTNF_R2_ (▴). Where indicated, MEF TNFR2-Fas were pretreated with the ligand/receptor stabilizing monoclonal antibody 80M2 (open symbols; 1 µg/ml; 30 min) before TNF treatment. Cell viability was determined by crystal violet staining after 24 hours (n = 3, shown are the mean values ± SEM).

TNF receptor selectivity of scTNF_R2_ and TNC-scTNF_R2_ was analyzed using TNFR1^−/−^/TNFR2^−/−^ mouse embryonic fibroblasts (MEF) stably expressing chimeric TNFR_ecd_-Fas_icd_ receptors that bind human TNF and trigger Fas-associated signaling pathways, e.g. apoptosis induction [21]. In contrast to human soluble recombinant TNF (huTNF), the TNFR2-selective TNF variants generated here induced no apoptosis in MEF TNFR1-Fas cells (Fig. 2A), verifying that both variants had highly reduced affinities for TNFR1 due to the mutations D143N/A145R. In contrast, TNC-scTNF_R2_ induced a strong apoptotic response in MEF TNFR2-Fas cells. Treatment with huTNF and scTNF_R2_ caused cell death only at high concentrations, which could be enhanced by addition of the cross-linking TNFR2-specific monoclonal antibody 80M2 (Fig. 2A) to similar values as obtained for TNC-scTNF_R2_ alone, thus in concert mimicking the action of memTNF [15]. Similar results were obtained using the human rhabdomyosarcoma cell line Kym-1 (Fig. 2B), which endogenously expresses both TNF receptors and is highly sensitive to TNF-induced cytotoxicity [22].

**Figure 3:**
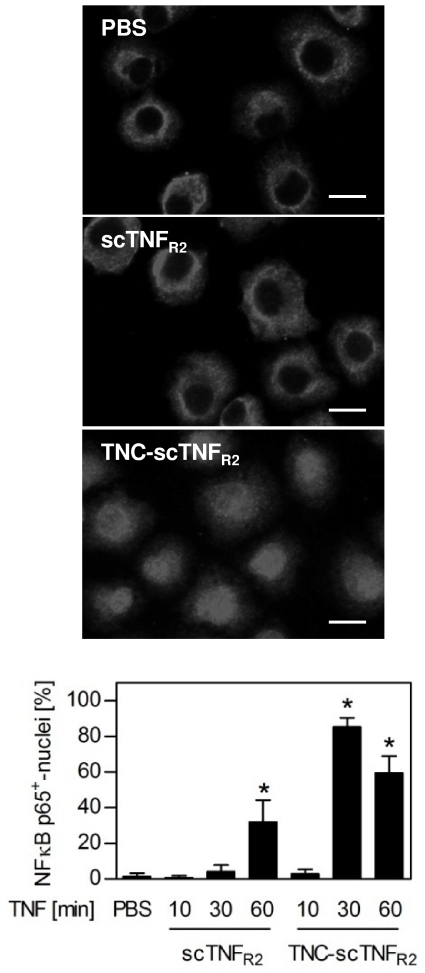
Comparison of the NFκB translocation after stimulation with scTNF_R2_ or TNC-scTNF_R2_. Kym-1 cells were stimulated with scTNF_R2_ or TNC-scTNF_R2_ (10 ng/ml) for 10, 30 or 60 minutes. Cells were fixed with 4% PFA, permeabilized with 0.1% Triton-X100 and stained with anti-NFκB p65 antibodies and Alexa-Fluor546-labeled secondary antibodies. Cell nuclei were visualized using DAPI. Shown are representative images at point of time 30 minutes (upper panel) and the quantification of the number of NFκB p65-positive nuclei. At least 200 cells were analyzed per condition. *p values less than 0.01 versus untreated controls were considered to be significant (n = 2; shown are the mean values ± SD; bar = 20 µm).

Using near to saturating concentrations (10 ng/ml), where both scTNF_R2_ and TNC-scTNF_R2_ showed maximum bioactivity in a long term bioassay (induction of cell death in Kym-1 cells as shown in 2B), we further investigated the kinetics of NFκB p65 nuclear translocation (Fig. 3). Whereas scTNF_R2_ stimulation only resulted in a significant translocation after 60 minutes with 32% of the cells showing nuclear NFκB, TNC-scTNF_R2_ stimulation resulted in a rapid NFκB translocation with an apparent peak at 30 minutes (85% NFκB^+^ cells), which was maintained at a high level for at least one hour (60% NFκB^+^ cells), further demonstrating the superior bioactivity of TNC-scTNF_R2_.

### TNC-scTNF_R2_ induces clustering of TNFR2

**Figure 4:**
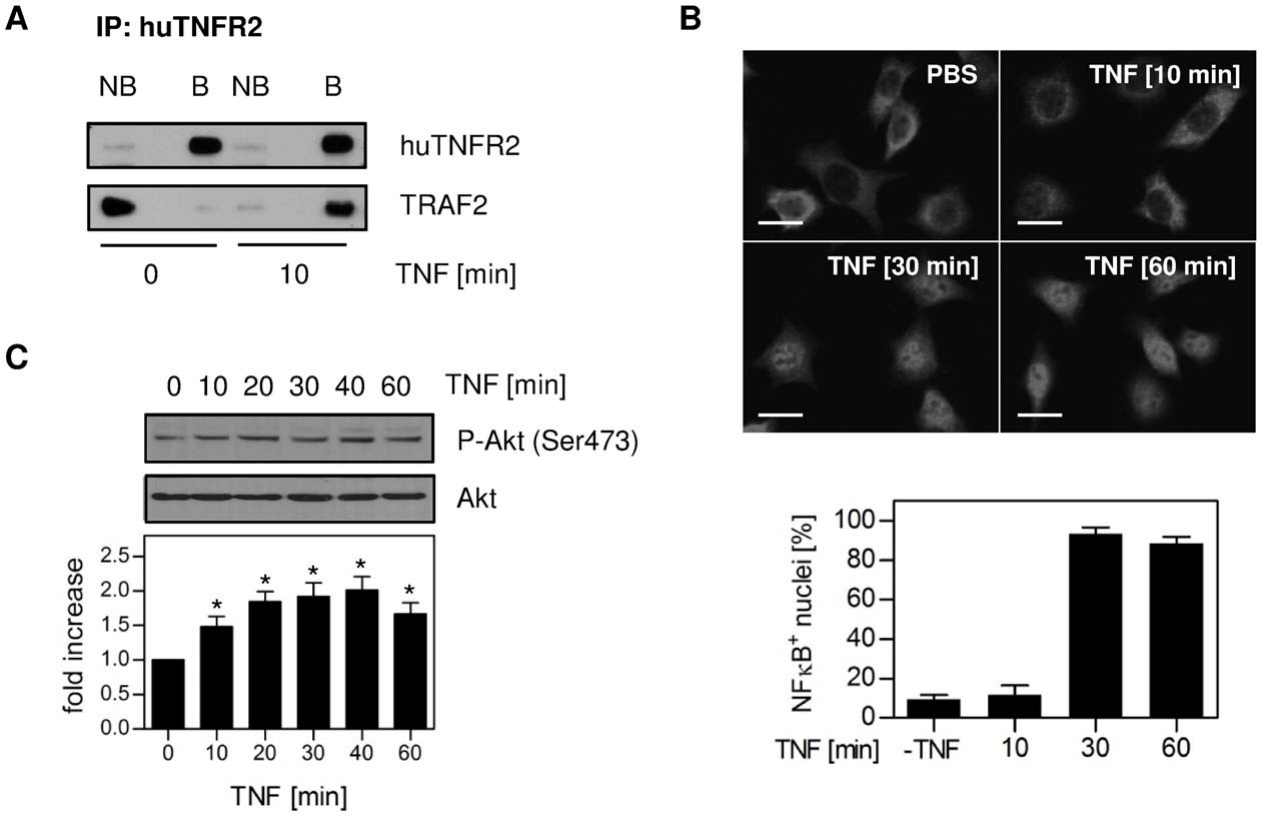
TNC-scTNF_R2_ induces TNFR2 signaling in R2 MEF. (**A–C**) R2 MEF were stimulated with TNC-scTNF_R2_ (20 ng/ml) for the indicated times. (**A**) TNFR2 was immunoprecipitated using MR2-1 antibodies and protein G agarose. The precipitates were analyzed by immunoblot analysis using anti-huTNFR2 (HP9003) and anti-TRAF2 antibodies (NB = non-bound; B = bound). (**B**) Localization of NFκB p65 was visualized via immunofluorescence microscopy as shown in the upper panel and the number of nuclei showing NFκB translocation was quantified. At least 200 cells per experiment were analyzed (n = 3; shown are the mean values ± SEM of percent NFκB positive nuclei; bar = 20 µm). (**C**) Phospho-Akt (Ser473) levels in cell lysates were analyzed using immunoblot analysis. Akt was used as a loading control. Representative blot and bar graph show the quantification of the phospho-Akt (Ser473) band. *p values less than 0.05 versus untreated cells were considered to be significant (n = 3; shown are the mean values ± SEM).

To further analyze the bioactivity of TNC-scTNF_R2_ we used the human TNFR2 (huTNFR2) expressing cell line R2 MEF [23]. We recently demonstrated in these cells that upon activation of TNFR2 by soluble TNF in combination with 80M2, the adaptor protein TRAF2 is recruited to the receptor and the classical NFκB pathway is activated [23]. Here we demonstrate that stimulation of R2 MEF with TNC-scTNF_R2_ results in co-immunoprecipitation of TRAF2 with TNFR2 (Fig. 4A) and activation of the classical NFκB pathway (Fig. 4B). In addition, the PI3K-PKB/Akt pathway was activated, as evident by the increased phosphorylation level of PKB/Akt after TNC-scTNF_R2_ stimulation (Fig. 4C).

**Figure 5:**
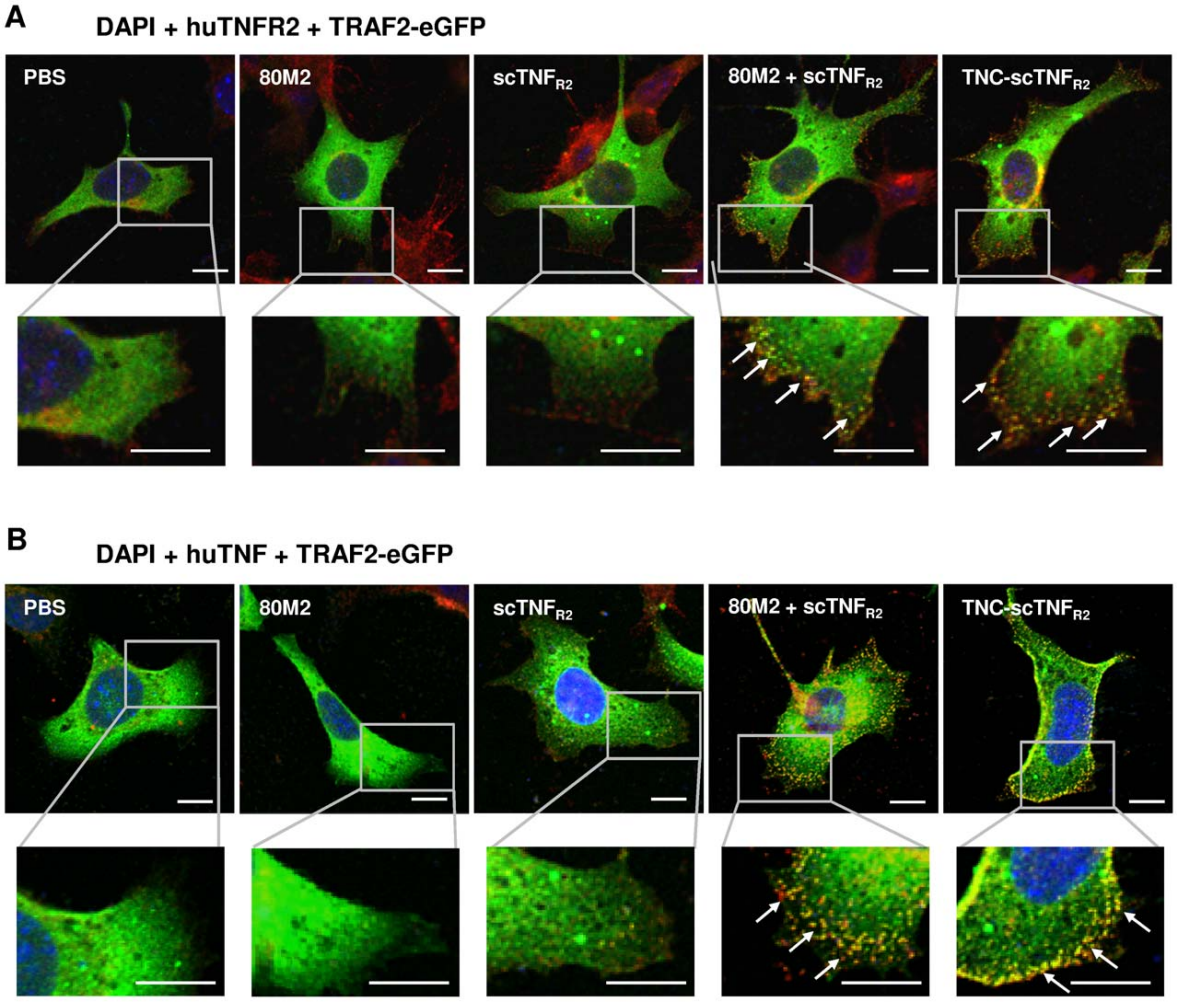
TNC-scTNF_R2_ induces formation of TNFR2 signaling complexes. (**A,B**) R2 MEF were transfected with pTRAF2-eGFP. After 24 hours, cells were incubated with or without 80M2 (1 µg/ml) for 5 minutes on ice and subsequently incubated with scTNF_R2_ or TNC-scTNF_R2_ (10 ng/ml) for 10 minutes at 37°C. Then cells were fixed with 4% PFA, permeabilized with 0.1% Triton X-100 and localization of huTNFR2 (**A**) or huTNF (**B**) was detected with specific antibodies and Alexa-Fluor546-labeled secondary antibodies. Cell nuclei were visualized using DAPI. Pictures are optical sections obtained by confocal fluorescence microscopy (bar = 10 µm). White arrows indicate areas of colocalization.

The enhanced activation of NFκB (Fig. 3) suggests a more efficient and/or faster formation of TNFR2 signaling complexes by TNC-scTNF_R2_. To confirm this, we investigated the ligand-induced recruitment of TRAF2, the essential cytosolic signal transducer of TNFR2, to the membrane using immunofluorescence analysis of a TRAF2-eGFP reporter (Fig. 5). Unstimulated R2 MEF expressing TRAF2-eGFP showed a homogenous cytosolic distribution of the reporter. As expected [23], cross-linking of TNFR2 by 80M2 resulted in a spotted appearance of the TNFR2 signal at the plasma membrane, yet did not induce colocalization with the TRAF2-eGFP signal (Fig. 5A). Similar, stimulation with scTNF_R2_ alone did not result in a detectable colocalization of the TRAF2-eGFP signal with either huTNFR2 (Fig. 5A) or scTNF_R2_ itself, stained with antibodies against human TNF (huTNF; Fig. 5B) during the observation period (10 min). In contrast, scTNF_R2_ in combination with 80M2 induced TNFR2 clustering and TRAF2 recruitment, apparent as a spotted appearance of huTNFR2 (Fig. 5A) or huTNF (Fig. 5B) signal colocalizing with TRAF2-eGFP. This finding indicates TNFR2 signaling complex formation [23]. In contrast to scTNF_R2_, TNC-scTNF_R2_ alone was sufficient to rapidly induce TNFR2 clustering and TRAF2 recruitment and thus efficient formation of TNFR2 signaling complexes (Fig. 5A and B). Summarizing, TNC-scTNF_R2_ showed superior bioactivity compared to scTNF_R2_ and was able to efficiently activate TNFR2 in distinct cell lines.

### TNC-scTNF_R2_ preserves neurons from superoxide-induced cell death

TNFR2 signaling has been associated with protection of neurons against cytotoxic insults [7], [8], [9]. Potential neuroprotective properties of TNC-scTNF_R2_ were investigated using the human cell line LUHMES, which has been shown to display typical biochemical and morphological features of human dopaminergic neurons [17], [18], [19], [20]. Differentiated LUHMES cells developed long dendrites, formed a neural-like network and expressed typical neuronal markers such as β-III-tubulin, α-synapsin, microtubule-associated protein 2 and neurofilament [18], [19] (data not shown).

**Figure 6:**
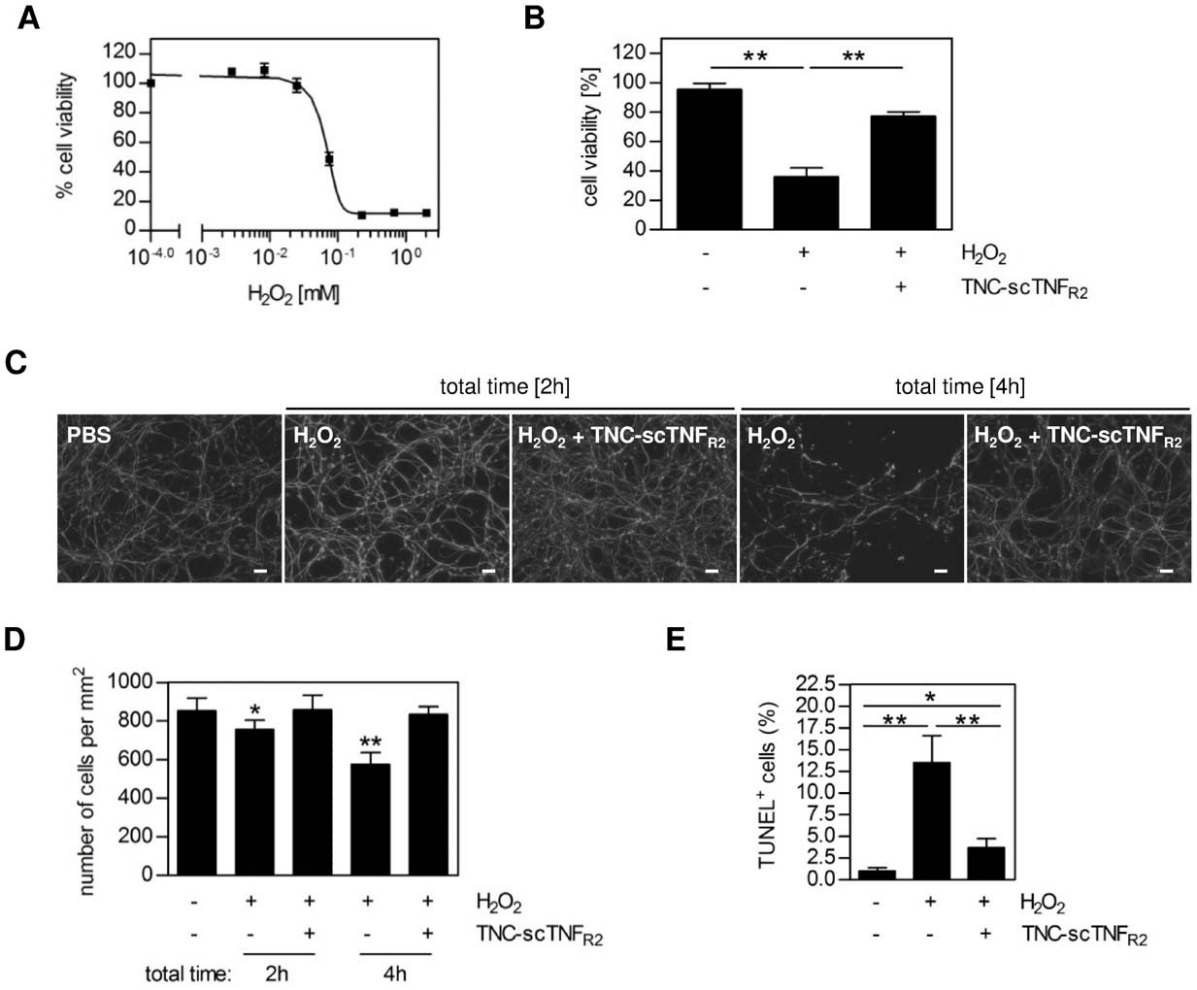
TNC-scTNF_R2_ induces neuroregeneration after H_2_O_2_-induced oxidative stress. (**A**) Differentiated LUHMES cells were incubated with different concentrations of hydrogen peroxide (H_2_O_2_) for one hour. Then cells were washed with medium und cultivated for additional 24 hours. Cell viability was measured using the MTT assay (n = 3; shown are the mean values of triplicate determinations ± SEM). (**B–E**) LUHMES cells were stimulated with H_2_O_2_ (100 µM). After one hour the cells were washed with medium und regenerated for the indicated time intervals in medium with or without TNC-scTNF_R2_ (100 ng/ml). (**B**) LUHMES cells were regenerated for 24 hours and cell viability was measured using the MTT assay (n = 3, shown are the mean values ± SEM). (**C**) Cells were regenerated for one or three hours, fixed with 4%PFA, permeabilized with 0.1% Triton-X100 and β-III-tubulin was detected with specific antibodies. Cell nuclei were visualized using DAPI. Pictures are projections of eight optical sections (0.4 µm; bar = 50 µm). (**D**) Number of cells was determined by counting the nuclei (DAPI staining). (**E**) Cells were regenerated for one hour and apoptotic cells were identified by terminal deoxynucleotidyl transferase (TdT)-mediated dUTP-FITC nick end labeling (TUNEL). (D,E) At least 10 different image sections containing a minimum of 500 cells were used to determine the number of total and TUNEL-positive cells. *p values less than 0.05 (** p-value less than 0.001) were considered to be significant (n = 2, shown are the mean values ± SD).

Short hydrogen peroxide (H_2_O_2_) exposure was used to induce cell death *in vitro*, simulating oxidative stress, a process that contributes to the damage in neurodegenerative diseases *in vivo*. To investigate the sensitivity of LUHMES cells to H_2_O_2_-induced cell death, the cells were incubated for one hour with the toxic chemical, then medium was exchanged to remove H_2_O_2_ and cells were cultivated for additional 24 hours. LUHMES cells started to die at H_2_O_2_ concentrations above 20 µM, with a half maximal effective concentration (EC50) of approximately 75 µM (Fig. 6A). Addition of TNC-scTNF_R2_ one hour after exposure to a toxic concentration of H_2_O_2_, counteracted the H_2_O_2_-triggered cell death pathway such that nearly 80% of the cells survived the treatment, compared to 30% survival without TNC-scTNF_R2_ (Fig. 6B).

To investigate the protective effect of TNC-scTNF_R2_ in detail, LUHMES cells were fixed two or four hours after starting the H_2_O_2_-treatment and the β-III-tubulin content (Fig. 6C) as well as cell number (Fig. 6D) were determined by immunofluorescence analyses. Already two hours after H_2_O_2_ exposure, a significant decrease in the number of cells was noted, which was further enhanced after four hours. In contrast, no significant loss of cells was observed at two and four hours in the samples treated with TNC-scTNF_R2_ one hour after H_2_O_2_ exposure. The rapid H_2_O_2_-induced cell death is at least partially due to apoptosis induction, as revealed from TUNEL-staining of exposed cells (Fig. 6E). A reduced amount of TUNEL-positive cells after regeneration in presence of TNC-scTNF_R2_ further indicates that TNFR2 signaling rapidly interferes with the H_2_O_2_-induced apoptotic signal pathways.

### TNC-scTNF_R2_-mediated protection from superoxide induced cell death is dependent on PI3K-PKB/Akt signaling

**Figure 7:**
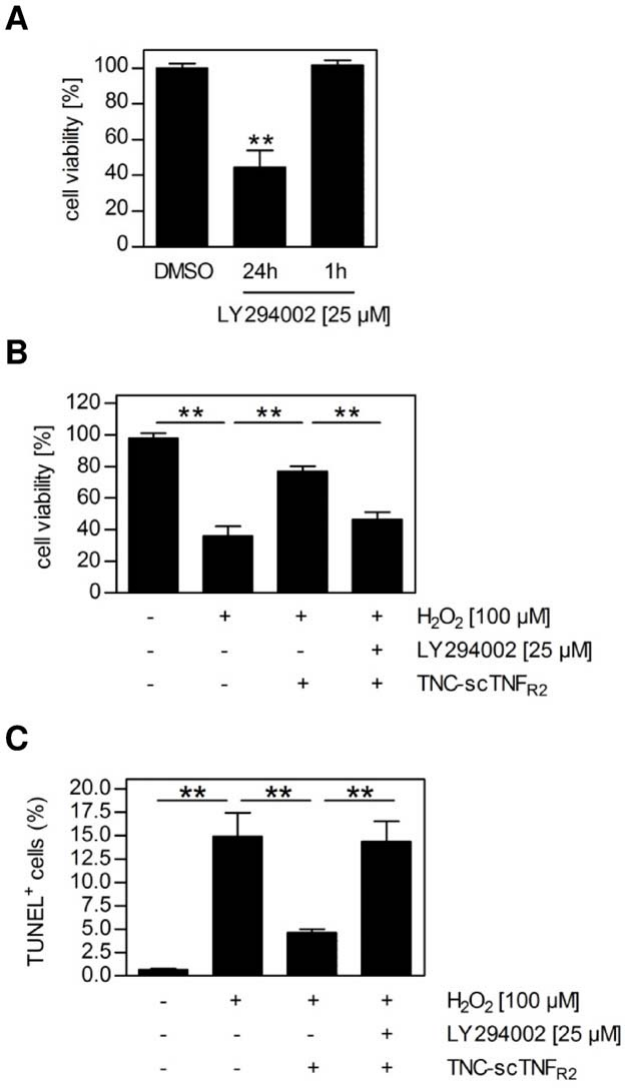
TNC-scTNF_R2_-mediated neuroregeneration is dependent on PI3K-PKB/Akt signaling. (**A**) LUHMES cells were stimulated with LY294002 (25 µM) for either 24 hours or for 1 hour. If stimulated for 1 hour, medium was exchanged to differentiation medium and cells were cultivated for further 23 hours. Cell viability was measured using the MTT assay (n = 3; shown are the mean values ± SEM). (**B,C**) LUHMES cells were stimulated with hydrogen peroxide (H_2_O_2_; 100 µM) with or without LY294002 (25 µM). After one hour the cells were washed with medium und regenerated in medium with or without TNC-scTNF_R2_ (100 ng/ml). (**B**) Cells were incubated for 24 hours and cell viability was measured using the MTT assay (n = 3, shown are the mean values ± SEM). (**C**) Cells were regenerated for one hour, fixed with 4% PFA and apoptotic cells were identified by terminal deoxynucleotidyl transferase (TdT)-mediated dUTP-FITC nick end labeling (TUNEL). At least 5 different image sections containing a minimum of 250 cells were used to determine the number of total and TUNEL-positive cells (n = 3, shown are the mean values ± SEM). **p values less than 0.001 were considered to be significant.

PKB/Akt exerts anti-apoptotic effects [24] and mediates cell protection in neurodegenerative diseases [25], [26], [27]. Furthermore, preconditioning via TNFR2-signaling spared primary neurons from glutamate-induced excitotoxicity *in vitro* in a PI3K-PKB/Akt-dependent manner [7], [8]. In accordance with these data, we could show that the protective effect mediated by TNC-scTNF_R2_ is dependent on PKB/Akt activity. First, whereas transient blocking of PI3K for one hour with the specific inhibitor LY294002 did not influence neuronal viability, persistent blocking of PI3K activity resulted in a dramatic decrease in cell viability after 24 hours of otherwise untreated neuronal cells (Fig. 7A), verifying the general importance of PI3K-PKB/Akt signaling for neuronal cell survival [18]. Second, upon oxidative stress already a transient inhibition of PI3K drastically affected protection by TNFR2 stimulation, as evident from reduced cell viability after 24 hours (Fig. 7B) and the elevated number of TUNEL-positive cells one hour post TNC-scTNF_R2_ treatment (Fig. 7C). This further demonstrates that activation of the PI3K-PKB/Akt pathway via TNFR2 results in anti-apoptotic signaling, thereby preventing the cell death induced by H_2_O_2_.

### TNC-scTNF_R2_ rescues neurons from catecholaminergic cell death

**Figure 8:**
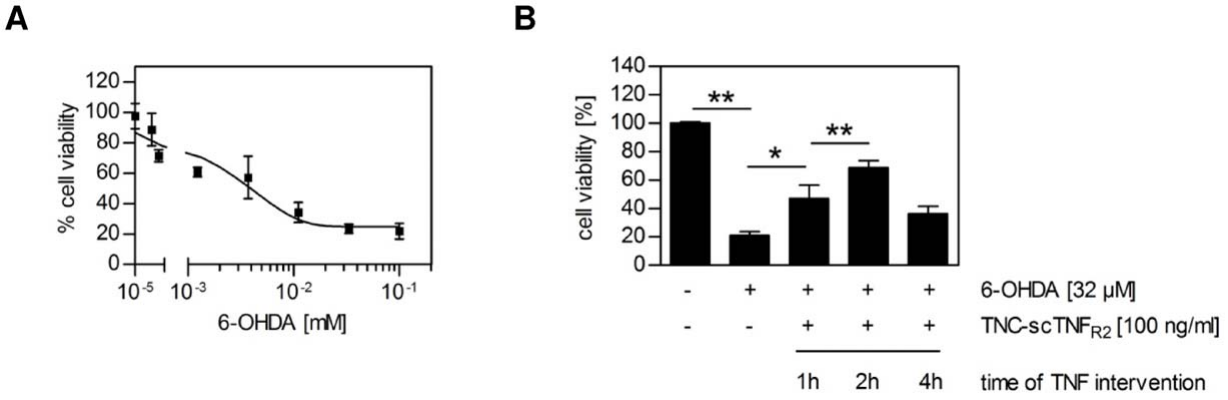
TNC-scTNF_R2_ protects neurons against catecholaminergic cell death. (**A**) Differentiated LUHMES cells were incubated with different concentrations of 6-hydroxydopamine (6-OHDA) for 20 hours. Cell viability was measured using the MTT assay (n = 3; shown are the mean values of triplicate determinations ± SEM). (**B**) LUHMES cells were stimulated with 6-OHDA (32 µM). Cells were stimulated with TNC-scTNF_R2_ (100 ng/ml) one, two or four hours after 6-OHDA addition and incubated for a total time period of 20 hours. Cell viability was measured using the MTT assay (n = 3, shown are the mean values ± SEM). *p values less than 0.05 (** p-value less than 0.001) were considered to be significant.

LUHMES cells differentiate into neurons with a dopaminergic phenotype [18]. The neurotoxin 6-hydroxydopamine (6-OHDA) is widely used as an oxidative stress *in vitro* model, which has relevance for PD. 6-OHDA generates free radicals and selectively induces cell death in catecholamine-containing neurons [28]. LUHMES cells were sensitive towards 6-OHDA-induced cell death with an EC_50_ of approximately 5 µM (Fig. 8A). Treatment of LUHMES cells with TNC-scTNF_R2_ one or two hours after exposure to a toxic concentration of 6-OHDA, counteracted the cell death pathway such that more than 50% of the cells survived the treatment, compared to 20% survival without TNC-scTNF_R2_ (Fig. 8B), further demonstrating the protective role of TNFR2 activation in oxidative stress-induced cell death.

## Discussion

Signaling via TNFR2 has been implicated in protection and regeneration of tissues, such as pancreas, liver, heart and brain [6]. Accordingly, TNF variants selectively activating TNFR2 could be a useful therapeutic regimen in a variety of diseases, including heart failure, autoimmune and neurodegenerative diseases. Towards this aim, here we describe a human TNFR2-selective TNF ligand with memTNF-mimetic activity (TNC-scTNF_R2_) that rescues differentiated neurons from cell death post insultem, i.e. under conditions that reflect the time span of a potential therapeutic intervention, for example to limit ischemic lesions after stroke.

Soluble TNF is a strong mediator of inflammation, predominantly through TNFR1, which is efficiently activated by the membrane-bound as well as the soluble form of TNF in the picomolar range [15]. Whereas TNFR2 binds soluble TNF as well as memTNF, it is only fully activated by the latter [15]. Therefore, potential TNFR2-specific therapeutics have to comply with two basic requirements: mimicry of memTNF function and receptor selectivity in order to avoid dose limiting severe TNFR1-related inflammatory responses. Fusion of the trimerization domain of tenascin C to a TNFR2-selective scTNF was shown in a previous study to exert memTNF-like activity [16]. We generated a fully human, optimized version of this fusion protein suitable to be further developed into a therapeutic.

The HPLC-SEC and SDS-PAGE data indicate that for scTNF_R2_ the majority of the molecules were present in monomeric form, i.e. resembling a soluble trimeric TNF, whereas the TNC-scTNF_R2_ assembled into functional multimers, most likely trimers, thus resembling a nonameric TNF molecule. The introduced double mutation D143N/A145R leads to a complete loss of binding to TNFR1 [12]. Therefore, as demonstrated in an apoptosis assay with a cell line selectively expressing TNFR1 (MEF TNFR1-Fas), TNC-scTNF_R2_ did not activate TNFR1. In contrast, TNC-scTNF_R2_ efficiently activated TNFR2, indicating that the oligomeric TNF mutein is able to mimic memTNF. As a consequence, TNC-scTNF_R2_ efficiently induced nuclear translocation of NFκB p65 in Kym-1 cells within 30 minutes, whereas stimulation with scTNF_R2_ only resulted in a marginal translocation after 30 minutes and a partial and delayed response at 60 minutes in Kym-1 cells. In these cells, this weak and late signal seemed to be strong enough to induce endogenous expression and secretion of TNF, which in an autocrine manner activated TNFR1 and subsequently the apoptotic signaling-cascade leading to Kym-1 cell death (Fig. 2C), as shown previously [22], [29].

Receptors of the TNF family are activated by ligand-mediated oligomerization [30] and efficient signal initiation requires the formation of larger ligand/receptor complexes [21], [31]. In previous studies, the initiation of cluster formation of TNFR2 was found to be dependent on exogenous cross-linking of the ligand-receptor complexes and correlated with the particular signaling strength [21]. Using the recently introduced huTNFR2-expressing cell line R2 MEF [23], the superior bioactivity of the multimeric TNC-scTNF_R2_ was further evident from rapid formation of TNFR2 signaling complexes, which can be readily visualized by fluorescence microscopy of TRAF2 recruitment. Summarizing, the soluble TNC-scTNF_R2_ ligand mimics memTNF-like activation of TNFR2 and can be used to selectively induce TNFR2 signaling *in vitro* and *in vivo*.

Parkinson's disease is a neurological disorder, which is characterized by the loss of dopamine-producing neurons in the substantia nigra causing reduced dopamine levels in the striatum [32]. This disease [33], like a variety of other neurodegenerative diseases, including Alzheimer's disease [34], amyotrophic lateral sclerosis [33] and conditions such as ischemia and excitotoxicity [35], has been associated with mitochondrial dysfunction and oxidative damage mediated by excessive production and exposure of cells to reactive oxygen species (ROS) resulting in neuronal death [36]. Oxidative stress can be simulated *in vitro* by brief exposure of cultured neurons to H_2_O_2_, resulting in the induction of cell death via the mitochondrial apoptosis pathway [37], [38]. In addition, neurotoxins, such as 1-methyl-4-phenyl-1,2,3,6-tetrahydropyridine (MPTP) or 6-OHDA mimic many of the hallmark characteristics of PD and are widely used as an oxidative stress model for PD [39], [40].

Preconditioning cells via TNFR2 signaling protected primary cortical neurons from excitotoxic cell death in an NFκB-dependent manner [7], [8]. In contrast, we activated TNFR2 by TNC-scTNF_R2_ subsequent to the toxic stimulus. Interestingly, under these conditions, simulating a therapeutic intervention, a strongly increased survival was noted, too, indicating that TNFR2 signaling can interfere with an already activated cell death program and promote cell survival.

Several pathways activated by TNFR2 mediate protective or regenerative responses. In particular, PKB/Akt-dependent activation of NFκB induces expression of anti-apoptotic and/or neurotrophic factors [7], [41]. In addition, PKB/Akt signaling directly exerts anti-apoptotic effects [24] and is involved in mediating cell protection in neurodegenerative diseases [25], [26], [27]. Since TNFR2 activation by TNC-scTNF_R2_ subsequent to the toxic exposure to H_2_O_2_ resulted in a fast protective response in a PKB/Akt-dependent manner, we assume that the protective effect is not dependent on *de novo* gene expression. Indeed, PKB/Akt has been shown to interfere with early apoptotic signals [42] and thus could mediate protection by directly interacting with components involved in the apoptotic signal pathway.

The molecular mechanisms of PKB/Akt-mediated survival from H_2_O_2_-induced toxicity in the neuronal cells studied here are not known yet. However, several components of the apoptotic machinery share the PKB/Akt consensus phosphorylation sequence (RXRXXS/T), e.g. pro-death components such as Bad, caspase-9 and caspase-8 or pro-survival factors like Bcl-2, cIAP and xIAP [42]. PKB/Akt therefore could regulate apoptosis by either inhibiting cell death or promoting cell survival. In particular, phosphorylation of Bad by PKB/Akt induces the dissociation of Bad from Bcl-X_L_, which then can promote cell survival. In addition, PKB/Akt was reported to directly block cell death after mitochondrial cytochrome C release, e.g. by phosphorylating caspase-9 at serine 196 [43] thereby inactivating the caspase. PKB/Akt therefore could potentially also interfere with the apoptotic pathway even at this rather late stage.

The mechanisms of the TNFR2-mediated protection from 6-OHDA-induced cell death are not characterized yet. However, Jordán et al. [44] present evidence that Bcl-X_L_ seems to prevent mitochondrial multiple conductance channel opening, cytochrome c release and caspase-3 like activity following 6-OHDA treatment. Therefore similar mechanisms could contribute to the protection of TNC-scTNF_R2_ against 6-OHDA-induced cell death as described for protection against H_2_O_2_-induced cell death, e.g activation of Bcl-X_L_ through phosphorylation of Bad.

In conclusion, we constructed a human TNFR2-selective TNF ligand possessing memTNF-mimetic activity and show that this molecule can rescue differentiated neurons from cell death subsequent to a toxic exposure, i.e. under conditions that reflect the time span of a potential therapeutic intervention. Our data, together with clear evidence that TNFR2 signaling promotes tissue regeneration in several organs [6], [10], indicate that selective activation of TNFR2 signaling may be a promising approach to treat various diseases, including autoimmune and neurodegenerative diseases. Being the first fully human TNFR2 agonist, TNC-scTNF_R2_ is suitable not only for further studies on the protective and regenerative mechanisms of TNFR2 signaling in different disease models but represents a prototype of a TNFR2-selective therapeutic which is in principle applicable in man as a novel therapeutic approach to promote tissue homeostasis and regeneration.

## Materials and Methods

### Antibodies and chemicals

The TNFR2 specific antibody 80M2 has been described [15]. LY294002 and the antibodies against NFκB p65, TRAF2, Akt and phospho Akt (Ser473) were from Cell Signaling Technology (Boston, MA). The antibody anti-6-his was from Biovision (San Francisco, CA), the antibodies against huTNFR2 (HP9003; MR2-1) were from Hbt (Uden, The Netherlands), the antibody against huTNF (AF-210-NA) was from R&D Systems (Wiesbaden, Germany) and the antibody against β-III-tubulin was purchased from Acris Antibodies (Hiddenhausen, Germany). Secondary antibodies coupled to horseradish peroxidase (HRP) and Alexa-Fluor488 or Alexa-Fluor546 were from Jackson ImmunoResearch (Suffolk, UK) and Invitrogen (Karlsruhe, Germany), respectively. DAPI, MTT (3-[4,5-dimethyl-thiazol-2-yl]-2,5-diphenyl tetrazolium bromide), 6-hydroxydopamine (6-OHDA) and H_2_O_2_ (hydrogen peroxide) were from Sigma-Aldrich (Steinheim, Germany). All other chemicals were of analytical grade.

### Cloning of scTNF_R2_ and TNC-scTNF_R2_

The TNFR2-selective scTNF_R2_ (D143N/A145R) and the human tenascin C (TNC) domain (aa 110–139) were synthesized with optimized eukaryotic codon usage (Geneart, Regensburg, Germany). The sequence of scTNF_R2_ was subcloned in frame into the expression vector pSecTag A (Invitrogen) containing an IgG secretion signal. Then TNC-scTNF_R2_ was generated by cloning of the TNC domain sequence to the N-terminal end of the scTNF_R2_ construct. All vector sequences were verified by sequencing.

### Cell culture

Immortalized mouse fibroblasts generated from TNFR1^−/−^/TNFR2^−/−^ mice and stably transfected with human TNFR1-Fas (MEF TNFR1-Fas) or TNFR2-Fas (MEF TNFR2-Fas) [21] or with human TNFR2 (R2 MEF) [23] have been described elsewhere. HEK293T cells and mouse fibroblasts were grown in RPMI1640 medium (Invitrogen) supplemented with 5% (v/v) heat-inactivated FCS (PAN Biotech, Aidenbach, Germany) and 100units/ml penicillin and 100 µg/ml streptomycin (Invitrogen). Kym-1 cells were cultivated in RPMI1640 medium supplemented with 10% (v/v) heat-inactivated FCS and penicillin/streptomycin. LUHMES cells were cultivated according to Schildknecht et al. [19].

### Production of scTNF_R2_ and TNC-scTNF_R2_

HEK293T cells were transfected with scTNF_R2_ or TNC-scTNF_R2_ expression constructs, using lipofectamine 2000 (Invitrogen). Stable cells were selected with Zeocin (Invitrogen). For protein production, cells were expanded in T175 culture flasks. When grown to 90% confluence, cells were cultivated for a total of 8 days with Opti-MEM (Invitrogen, USA). Medium was changed after 2 days. Supernatant was centrifuged (720*g*; five minutes) and stored at 4°C. TNF variants were purified by immobilized metal ion chromatography (IMAC). For this purpose, supernatant was loaded onto a column containing Ni-NTA agarose (Qiagen, Hilden, Germany) and unbound proteins were washed away using IMAC wash buffer (50 mM sodium-phosphate-buffer, 10–30 mM imidazole). Bound proteins were eluted with IMAC elution buffer (50 mM sodium-phosphate-buffer, 100 mM imidazole) in fractions of 1 ml. The purity of the protein in the eluted fractions was analyzed by SDS-PAGE and pure fractions were pooled and dialyzed (cut-off 4–6 kDa, Roth) against PBS overnight at 4°C. Protein concentration was determined by measuring the absorbance at 280 nm.

### SDS-PAGE and Immunoblot

Cells were lysed in homogenization buffer (10 mM HEPES pH 7.5, 1.5 mM MgCl_2_, 1.5 mM KCl, 1% NP-40, 0.2 mM PMSF, 20 mM ß-glycerophosphate and 100 µM Na_3_VO_4_) at 4°C for 30 min. Lysates were centrifuged (2 min at 9600 g) and protein concentration of supernatants were determined using the BCA method (Pierce, Bonn, Germany). 20 µg total protein or 2 µg (SDS-PAGE) and 1 µg (immunoblot) respectively of the TNF muteins were denatured in Laemmli buffer and resolved by 8% SDS-PAGE (100 V; 90 minutes). For Coomassie staining of proteins the gel was incubated in Coomassie staining solution for 60 minutes at room temperature and destained. For immunoblot analyses the proteins were transferred onto nitrocellulose membranes (semidry blot; 1.5 mA/cm^2^ gel for 90 minutes). Non-specific protein binding was blocked with 5% skimmed milk powder solution in PBS/0.1% Tween20 for 30 minutes at room temperature and the membrane was incubated overnight at 4°C using specific antibodies. After incubation with anti-rabbit HRP-conjugated secondary antibodies for 90 minutes at room temperature the signals were detected by enhanced chemiluminescence (Super Signal, Pierce, Rockford, IL).

### Immunoprecipitation

Approximately 5×10^6^ R2 MEF were seeded in T75 cell culture flask (Greiner) and cultivated overnight. Cells were stimulated as indicated and homogenized with 200 µl IP lysis buffer (10 mM TRIS pH 7.4, 100 mM KCl, 1 mM EDTA, 1 mM EGTA, 0.5% Triton X-100, 0.5% NP-40, 100 µM Na_3_VO_4_, 0.2 mM PMSF, 20 mM ß-glycero-phosphate, Roche complete protease inhibitor cocktail) for 30 minutes on ice. The lysate was centrifuged (2 minutes; 9600*g*; 4°C) and the supernatant was incubated with 5 µg TNFR2-specific antibody MR2-1 (Hbt) for 2 hours at 4°C. The immunocomplexes were precipitated with protein G agarose (75 µl; Pierce) for two hours at 4°C. The precipitates were washed four times with IP washing buffer (10 mM TRIS pH 7.4, 100 mM KCl, 1 mM EDTA, 1 mM EGTA, 1% NP-40) and dissolved in 100 µl PBS, supplemented with Laemmli buffer. To elute bound proteins, the precipitates were boiled for five minutes at 95°C. 30 µl of the eluted proteins were separated by SDS-PAGE and analyzed by immunoblot analysis.

### HPLC

The oligomerization state of the TNF variants under native conditions was analyzed by HPLC-SEC. 50 µl sample (0.1 to 0.5 mg/ml) was applied to a BioSep-SEC-S2000 7.8×300 column (Phenomenex, Aschaffenburg, Germany) equilibrated with PBS and eluted at a flow rate of 0.5 ml/min. For determining the size of recombinant proteins, standard proteins (29 kDa, carbonic anhydrase; 67 kDa, bovine serum albumin (BSA); 200 kDa, β-amylase; 443 kDa, apoferritin; 669 kDa, thyroglobulin) were run under the same conditions.

### Cell survival assays

#### Crystal violet

Mouse fibroblasts or Kym-1 cells (1×10^4^ cells/well) were grown in 96-well flat bottom cell culture plates overnight. If indicated, the cells were stimulated with 80M2 (1 µg/ml) for 30 minutes. Then the cells were incubated with different concentrations of TNF variants for 24 hours at 37°C. The cells were washed with PBS and incubated with crystal violet (20% methanol; 0.5% crystal violet) for 20 minutes to stain viable cells. The dye was washed away under rinsing water and the cells were air-dried. Crystal violet was resolved with methanol and the optical density at 550 nm was determined. Each sample was analyzed in triplicates and data were analyzed using the software Microsoft Excel and GraphPad Prism 4.

#### MTT

Fully differentiated LUHMES cells cultivated in 96-well plates were stimulated with H_2_O_2_ with or without LY294002 (25 µM). After one hour cells were washed with medium and regenerated in medium with or without TNC-scTNF_R2_ (100 ng/ml). After 24 hours, the medium was removed carefully and 60 µl MTT solution (1,25 mg/ml) diluted 1∶6 in DMEM (1% FCS) was added. Cells were incubated for 90 minutes at 37°C. Then 150 µl acidic isopropanol was added and cells were dissolved. Absorbance was measured at 550 nm.

Differentiated LUHMES cells were stimulated with 6-OHDA. After 1, 2 or 4 hours TNC-scTNF_R2_ (100 ng/ml) was added and cells were cultured for a total time of 20 hours. Cell viability was determined as described above. Each sample was analyzed in triplicates and cell viability was calculated using the software Microsoft Excel and GraphPad Prism 4.

#### TUNEL

Fully differentiated LUHMES cells cultivated on cover slips were stimulated with H_2_O_2_. After one hour, the cells were washed with medium and regenerated for the indicated times in medium with or without TNC-scTNF_R2_ (100 ng/ml). At point in time zero, the cells were washed with PBS, fixed with PBS/4%PFA for 20 minutes at 37°C and permeabilized with PBS/0.1% TritonX-100 for five minutes at room temperature. Labeling with the TUNEL reaction mix was performed due to the recommendations of the manufacturer (Roche Applied Science, Mannheim, Germany). Coverslips were mounted with Fluoromount G (Southern Biotech, Birmingham, AL). The fluorescence was analyzed by wide-field fluorescence microscopy (CellObserver, Carl Zeiss).

### Fluorescence microscopy

For immunofluorescence analyses LUHMES cells were cultivated on PLO-fibronectin-coated cover slips and differentiated for 72 hours as described in [19]. R2 MEF and Kym-1 cells (3×10^4^ cells/well) were cultivated overnight on Lab-Tek 8-well chamber glass slides (Thermo, Schwerte, Germany) coated with 10 µg/ml poly-D-lysine (Sigma) for one hour at 37°C. If indicated in the legend, R2 MEFs were transiently transfected with pTRAF2-eGFP expression constructs using lipofectamine 2000 (Invitrogen) according to the manufacturer's recommendations. The next day, cells were stimulated with TNF muteins, as indicated, and fixed with PBS/4% PFA for 30 minutes on ice at point in time zero. Then cells were permeabilized with PBS/0.1% Triton-X100 for 10 minutes at room temperature. Unspecific binding sites were blocked with PBS/4% BSA for 30 min and cells were subsequently incubated with primary antibodies for 60 min followed by the incubation with appropriate fluorescence-labeled secondary antibodies for 45 min in PBS/2% BSA. After staining the nuclei with DAPI the cells were mounted with Fluoromount G (Southern Biotech). The labeled cells were analyzed by wide-field fluorescence microscopy (CellObserver with Colibri LED modules, Carl Zeiss, Jena, Germany) or by confocal laser scanning microscopy (LSM710, Carl Zeiss). The analysis of the obtained data and imaging was performed with the AxioVision Rel 4.7 (Carl Zeiss; CellObserver) or ZenLightEdition (Carl Zeiss; LSM710) software. Isotype or secondary antibodies control stainings were used to determine background fluorescence and to adjust the intensity of specific stainings.

### Quantification and statistical analysis

To quantify protein amounts, detected by immunoblot blot analysis, films were scanned and integrated optical densities were calculated using ImageJ. Integrated optical densities were corrected for background intensities. For quantification of nuclear translocation of NFκB in immunofluorescence experiments, the number of cells with NFκB positive nuclei of at least 200 cells per experiment and condition was determined.

Normal distribution was analyzed by Shapiro-Wilk normally test. Statistical analysis was performed by the Tukey range test. P<0.05 was considered significant.
